# Exploring the ability of self-report measures to identify risk of high treatment burden in chronic disease patients: a cross-sectional study

**DOI:** 10.1186/s12889-022-12579-1

**Published:** 2022-01-24

**Authors:** Ruth Hardman, Stephen Begg, Evelien Spelten

**Affiliations:** 1 La Trobe Rural Health School, La Trobe University, PO Box 199, Bendigo, Victoria 3552 Australia; 2 Sunraysia Community Health Services, 137 Thirteenth Street, Mildura, Victoria 3500 Australia

**Keywords:** Chronic diseases, Multimorbidity, Patient capacity, Treatment burden, Deprivation

## Abstract

**Background:**

Effective self-management of chronic health conditions is key to avoiding disease escalation and poor health outcomes, but self-management abilities vary. Adequate patient capacity, in terms of abilities and resources, is needed to effectively manage the treatment burden associated with chronic health conditions. The ability to measure different elements of capacity, as well as treatment burden, may assist to identify those at risk of poor self-management. Our aims were to: 1. Investigate correlations between established self-report tools measuring aspects of patient capacity, and treatment burden; and 2. Explore whether individual questions from the self-report tools will correlate to perceived treatment burden without loss of explanation. This may assist in the development of a clinical screening tool to identify people at risk of high treatment burden.

**Methods:**

A cross-sectional survey in both a postal and online format. Patients reporting one or more chronic diseases completed validated self-report scales assessing social, financial, physical and emotional capacity; quality of life; and perceived treatment burden. Logistic regression analysis was used to explore relationships between different capacity variables, and perceived high treatment burden.

**Results:**

Respondents (*n* = 183) were mostly female (78%) with a mean age of 60 years. Most participants were multimorbid (94%), with 45% reporting more than five conditions. 51% reported a high treatment burden. Following logistic regression analyses, high perceived treatment burden was correlated with younger age, material deprivation, low self-efficacy and usual activity limitation. These factors accounted for 50.7% of the variance in high perceived treatment burden. Neither disease burden nor specific diagnosis was correlated with treatment burden.

**Conclusions:**

This study supports previous observations that psychosocial factors may be more influential than specific diagnoses for multimorbid patients in managing their treatment workload. A simple capacity measure may be useful to identify those who are likely to struggle with healthcare demands.

**Supplementary Information:**

The online version contains supplementary material available at 10.1186/s12889-022-12579-1.

## Background

Living successfully with chronic health conditions (CHCs) requires effective self-management, including completing specific treatment tasks, lifestyle modifications and managing the physical, social and emotional impacts on one’s daily life [1–3]. Self-management abilities vary, and people who struggle with self-management are at greater risk of disease escalation, preventable hospitalisation, and mortality. Earlier identification of those at risk of poor self-management could enable targeted support to circumvent such outcomes.

Most risk prediction in chronic disease has focussed on quantifiable late-stage outcomes such as hospitalisation or mortality, using disease counts and biomarkers [4–7]. Although measures of self-management ability and patient engagement exist [8, 9], clinically usable measures to identify those likely to struggle with self-management have not been well-explored. This is despite copious literature describing the barriers to self-management [10–14].

Health professionals’ assessments of self-management ability, patient capacity and treatment burden can be at odds with the patient experience [15, 16]. Clinicians focus on biomedical status [16, 17] and perceived motivation [18] when assessing patient capacity, whereas patients consider resource constraints and social support levels to be more important [15, 19, 20]. Underestimation of treatment burden by health professionals has also been reported [20], who often focus only on adherence to specific treatment tasks. Treatment burden is a broader concept which is dependent on individual perception. It includes social, emotional, and financial aspects, as well as the difficulty in task management when one is ill, fatigued or in pain [15, 17, 21].

An assessment of self-management barriers informed by the patient, rather than the clinician perspective may better identify those people likely to struggle with self-management. Structuring a risk assessment tool is challenging given the multiple factors associated with poor self-management but using a capacity-burden model such as the Cumulative Complexity Model [22] can provide direction. In this model, successful CHC management relies on a balance between capacity and burden [22]. Capacity describes internal and external resources such as physical functioning, income, and social support. Burden, or workload, includes accessing healthcare, adhering to treatment recommendations, and maintaining a purposeful life. The perception of treatment burden will depend on individual capacity. A modest number of treatment tasks can be experienced as an overwhelming burden if capacity is insufficient, whilst patients with high levels of capacity may be able to cope with significant healthcare demands [23]. If there is inadequate capacity to service the burden, the patient may struggle with self-management. This can lead to condition deterioration (further reducing capacity) and treatment escalation (increasing treatment burden) – hence cumulative complexity [21, 22, 24].

Measuring capacity and burden has been recommended in order to discover those at risk of cumulative complexity [21, 24–26]. Self-report treatment burden tools have been developed [27–29], as well as assessments of capacity such as illness burden, financial and social capacity scales [30–32]. For patients with established multimorbidity, direct measurement of treatment burden has been recommended [33]. In our study, we have instead chosen to focus on capacity measurement for two reasons: first, it could be undertaken early, at the point of patient assessment or diagnosis, prior to treatment provision or self-management recommendations. Alerting the clinician to capacity constraints (thus limited ability to manage treatment burden) would direct them to simplify treatment demands and/or provide additional support. Secondly, measuring different elements of capacity could enable the clinician to pinpoint the specific barriers for that patient.

Our aim was to investigate the ability of different capacity domains to act as a ‘flag’ to identify those more likely to report high treatment burden. We intended to combine already validated self-report scales to see if they could provide an overall picture of capacity, and potentially act as a short screening measure usable in the clinic environment.

The aims of this study are:

1. To explore the correlations between established self-report tools that measure aspects of capacity, and treatment burden.
2. To discover whether selected individual questions from the self-report tools will correlate to treatment burden without loss of explanation.

We hope that this analysis may support the development of a clinically useful tool to identify people at risk of high perceived treatment burden.

## Methods

### Study design

This was a cross-sectional design involving the analysis of data from a survey undertaken both online and in a clinic population. Research was conducted in accordance with national ethics guidelines and approval was granted by the La Trobe University Human Research Ethics Committee (HEC number 19517).

The choice of screening tools was informed by the Cumulative Complexity Model [22] and other studies influenced by this model [23, 34–36].

### Recruitment and participants

Adults over the age of 18 with at least one chronic health condition were eligible to participate in the survey. The survey invited “anyone who has one or more chronic health conditions (e.g., arthritis, diabetes, chronic pain, heart or lung disease) that affect their daily life” to participate. Although the overall focus was on multimorbidity, with one tool developed specifically for multimorbid populations, a decision was made not to specify a certain number of health conditions since complexity may still occur when someone has only one health condition but a complex psychosocial environment [5, 6].

The onset of COVID-19 required a pivot from the original recruitment plan, which involved direct enrolment of participants from community health waiting rooms and activity groups using paper and ipad-based surveys. With services in lockdown and the switch to telehealth, we instead moved to conducting the survey both online and via post. The online survey was available from March to December 2020. It was placed on two patient advocate websites – Arthritis Australia and Diabetes Australia – as well as the website of the community health centre where the postal survey was run. The postal survey was sent in March 2020 to 400 clients who were registered with the chronic disease service of a large regional community health service in Victoria, Australia. Due to resource constraints related to COVID, we were unable to follow up non-respondents. Both paper and online surveys stated that the researchers were investigating ways to help health professionals support people with CHCs, that the survey was anonymous and voluntary and would take 15–20 min to complete. Informed consent was inferred based on completion of the survey.

### Survey measures

The focus in this study was to choose already validated generic (not disease-specific) tools which were short and simple enough to be used in a clinical setting. If the self-report tools proved useful, our intention was to eventually modify the survey to screen for risk in a chronic disease population. The survey was trialled with a convenience sample of researchers and their acquaintances, but since all self-report scales had been previously validated, further pilot testing was not undertaken.

#### Dependent variable

The primary dependent variable was perceived treatment burden, measured using the Multimorbidity Treatment Burden Questionnaire (MTBQ) [29]. This is a 10-item (plus three optional items) Likert scale measure which ranks the difficulty of healthcare tasks. We used all thirteen items since all were considered relevant in the Australian healthcare environment. The MTBQ has good internal consistency and was validated in a large multimorbid primary care population. It was chosen because it was a shorter and more simply worded tool than the other available treatment burden measures [27, 28], and our focus was on clinical usability. We calculated both a global MTBQ score as well as treatment burden ranking (none, low, medium, or high burden), following the scoring process described by Duncan et al. [29, 37].

#### Independent variables

The independent variables were chosen to cover key capacity domains. There are currently no validated tools to assess capacity in its entirety. Capacity describes the ability for a person to manage their treatment load in terms of their abilities and resources. It includes social support, socioeconomic resources, literacy, attitudes/beliefs, and level of mental/physical functioning [34, 36]. Since the aim was to trial established, clinically usable tools, we decided to include the following aspects of capacity: economic, social, personal, and physical.

To assess economic and social capacity, we used the Deprivation in Primary Care Questionnaire (DiPCare-Q) which consists of 16 yes/no questions assessing individual social, financial and health disadvantage [31]. This has good psychometric properties (ICC = 0.847); has been validated in a primary care chronic disease population [31, 38] and is correlated with treatment burden and quality of life [39]. Although this is a Swiss scale not previously used in Australia, the DiPCare-Q has been professionally translated into several languages including English. We were unable to find any other measures of individual deprivation [32, 40, 41] that had been validated in a primary care population. Following the instructions of Leiser et al. [38] we generated an overall DiPCare-Q index (ranging from 0 to 5.4), as well as a material (MatDCQ) and social (SocDCQ) deprivation score to use in analysis.

Personal capacity includes attitudes, beliefs, resilience, and self-efficacy. We chose to focus on self-efficacy for several reasons. Of the wide range of health attitudes and beliefs, self-efficacy stands out as a well-defined and strong psychological predictor across multiple outcomes associated with chronic health conditions [1, 2]. Whilst resilience is important, the concept is poorly defined, and current measures cross into several different capacity domains. Therefore, we used the short form Perceived Medical Condition Self-Management Scale (PMCSMS-4) [42] to assess personal capacity. This is a validated 4-item Likert scale (scored from 4 to 20), assessing self-efficacy for self-management of CHCs. The measure was chosen because it is not disease-specific, very short and judged to be more simply worded than comparable generic self-efficacy measures.

To assess physical capacity, we used the Disease Burden Impact Scale (DBIS) [30, 43]. This consists of a list of 25 possible medical conditions (plus the ability to report ‘other’ conditions). For each reported condition, the respondent uses a 5-point Likert scale to rate the interference in daily life caused by that condition. This has been found to be more predictive of quality of life than a disease count [43], and has been validated in a large multimorbid primary care population [30, 44]. It has also been correlated with the MTBQ [29] and the EQ-5D5L [43]. We followed Peters et al. [43] in modifying the original DBIS to include mental health and additional neurological diagnoses, and slightly reworded some terminology to increase understanding for the Australian audience. Although we recorded condition count, we did not analyse it as a separate variable, since the DBIS encompasses both CHC count and impact.

We also included the EQ-5D5L, a 5-item Likert scale plus VAS score (the VAS component was not used in the analysis). This is a widely used quality-of-life measure with good psychometric properties [45] which has previously been correlated with three of our chosen independent variables: the MTBQ [29], the DBIS [43] and the DiPCare-Q [39]. Because the Australian population norms for the EQ-5D5L have not been reported, we used the UK scoring algorithm to calculate a single index score. This process has been successfully applied in other Australian studies [46].

Finally, we included the presence of diabetes or a mental health diagnosis (as reported in the DBIS) as dichotomous variables, since they are the only specific conditions that have previously been associated with increased treatment burden [29, 39, 47].

#### Covariates

Our covariates were age and gender. Higher reported treatment burden has previously been correlated with younger age [29, 39, 47] and female gender [29]. Since one aim of this study was to identify the smallest number of variables needed to correlate with treatment burden, we only included covariates that have previously been associated with treatment burden and excluded those that might overlap with other capacity measures.

### Analysis

Scores for each of the self-report tools were calculated according to the instructions provided by the developer of each measure. All data was entered into SPSS version 25.0 for analysis. In our descriptive analysis, we aimed to compare our survey population to those populations in whom the self-report measures were initially validated. We also calculated Cronbach’s alpha for three of the self-report measures used to confirm reliability. We then undertook bivariate analysis across all variables of interest.

Our approach to multivariate analysis was informed by our aim to develop a simple screening tool. We therefore undertook logistic regression analysis, comparing high treatment burden to no/low/medium burden, since this would be easier to interpret in a time-poor clinical environment. Independent variables were selected based on whether they were significantly correlated with the dichotomous treatment burden variable in bivariate analysis, and we built a series of models to identify the best fit. Our plan was for each model to include a measure of physical, personal, economic, and social capacity, but, with a potential screening tool in mind, we wanted to minimise the number of self-report items that would be needed. Missing data was addressed by imputation using median score or commonest category, and sensitivity analysis was conducted to confirm that this did not influence the results.

## Results

### Descriptive analysis

#### Participant characteristics

183 surveys were returned – 80 postal (20% return rate) and 103 online. The population was 78% female with a mean age of 60.1 years. The online and postal populations differed, with the online respondents more likely to be younger (mean 53 yrs. compared to mean 68 yrs), female (91%) and living in a capital city. This reflects the fact that the postal survey was conducted in a rural setting amongst an older community health population. Only 30.4% of respondents were employed either full- or part-time, with the majority either retired from or unable to work due to health.

94% of participants reported more than two CHCs, with 45% reporting more than five. Recoding for some DBIS scores was required due to double scoring (when a condition was selected and then listed again under ‘other condition’) or when the condition was selected but the impact not rated. For double scoring, the higher score was included and the lower excluded and when the impact was not rated, a score of 1 (‘does not interfere’) was allocated. The median DBIS score was 15 (scores were positively skewed); this was comparable to Peters [43]. The most common condition grouping was musculoskeletal disorders (91.2% of respondents), followed by cardiovascular (56%) and mental health conditions (50%). Although these CHCs are all prevalent in the Australian population [48], the very high number of people reporting musculoskeletal conditions likely reflects the fact that online participation was largely via the Arthritis Australia portal.

Since neither the DiPCare-Q nor the MTBQ had been previously used in an Australian population, we confirmed acceptable reliability for both these scales (DiPCare-Q: KR-20 = 0.782; MTBQ: Cronbach’s α = 0.913) and for the EQ-5D5L (Cronbach’s α = 0.773) in our population. All scales had non-normal distributions, therefore we included median/IQR as well as mean/SD values for each variable. Demographic and descriptive data are presented in Table 1.

**Table 1:** Descriptive characteristics

Description	Value	Freq/mean/median	Percent/SD/IQR	Missing values
Age	Mean/SD	mean = 60.1	SD = 16.5	*n* = 3
Gender	Female	*n* = 143	78.1%	*n* = 3
Employment	Working (full/part)	*n* = 55	30.4%	*n* = 2
	Retired	*n* = 74	40.9%	
	Not working due to health	*n* = 34	18.8%	
	Other	*n* = 18	9.8%	
Number of conditions reported^a^	1	*n* = 11	6.0%	*n* = 1
	2–5	*n* = 89	48.6%	
	More than 5	*n* = 82	45.0%	
Condition type^b^	Musculoskeletal^1^	*n* = 166	91.2%	*n* = 1
	Cardiovascular^2^	*n* = 102	56%	
	Mental health^3^	*n* = 91	50%	
	Respiratory^4^	*n* = 55	30.2%	
	Diabetes	*n* = 36	19.8%	
DBIS score	Mean/SD	mean = 18.04	SD = 12.96	*n* = 1
	Median/IQR	median = 15	IQR = 17	
PMCSMS-4 score	Mean/SD	mean = 12.15	SD = 3.44	*n* = 2
	Median/IQR	median = 12.00	IQR = 5	
EQ-5D5L	Mean/SD	mean = 0.575	SD = 0.246	*n* = 5
	Median/IQR	median = 0.626	IQR = 0.341	
DiPCare-Q	Mean/SD	mean = 1.96	SD = 1.30	*n* = 5
	Median/IQR	median = 2.00	IQR = 2.00	
	MatDCQ: Mean/SD	mean = 0.89	SD = 0.965	
	MatDCQ: Median/IQR	median = 1	IQR = 2	
	SocDCQ: Mean/SD	mean = 2.36	SD = 1.17	
	SocDCQ: Median/IQR	median = 2	IQR = 1	
MTBQ	Median/IQR	median = 23.08	IQR = 35.58	*n* = 14
	MTBQ rank: none	*n* = 20	11.8%	
	MTBQ rank: low	*n* = 31	18.3%	
	MTBQ rank: medium	*n* = 31	18.3%	
	MTBQ rank: high	*n* = 87	51.5%	

^a^Based on the number of conditions selected on the DBIS. This may include several conditions of the same type, as listed below

^b^Number of people who reported one or more conditions under the following DBIS headings: 1 Musculoskeletal: Back pain/sciatica; Osteoarthritis; Osteoporosis; Rheumatoid arthritis; Other muscle/joint pain condition (e.g. fibromyalgia). 2 Cardiovascular: High blood pressure; High cholesterol; Angina/heart disease; Heart failure. 3 Mental health: Anxiety/depression; Other mental health (e.g. bipolar). 4 Respiratory: Bronchitis/COPD; Asthma

### Bivariate analysis

Univariate analysis confirmed that all scales had non-normal distribution, therefore non-parametric tests were employed for bivariate analysis. We conducted bivariate analysis on both the global MTBQ score (GMTBQ) and the dichotomous treatment burden variable (MTBQ-2) used in regression, but include only results for the categorical variable. Results are summarised in Table 2.

**Table 2:** Bivariate correlations

	Age	DBIS	PMCSMS-4	DiPCare-Q	EQ-5D5L	MTBQ-2 (Dependent)
**Age**	X	SR = 0.158^*^	SR = 0.267***	SR = − 0.229**	n.s. *p* = 0.079	MW = − 0.362***
**Gender**	MW = − 0.255**	n.s. *p* = 0.765	n.s. *p* = 0.279	n.s. *p* = 0.924	n.s. *p* = 0.711	Phi = 0.216**
**Disease burden measures (Physical capacity)**						
**DBIS score**	X	X	SR = −0.318***	SR = 0.313***	SR = − 0.534***	MW = 0.299***
**Has diabetes**	MW = 0.307***	MW = 0.208**	n.s. *p* = 0.497	n.s. *p* = 0.691	n.s. *p* = 0.606	n.s. p = 0.057
**Has mental health condition**	MW = −0.196**	MW = 0.420***	MW = −0.305***	MW = 0.361***	MW = − 0.277***	Phi = 0.337***
**Self-efficacy measures (Personal capacity)**						
**PMCSMS-4 score**	X	X	X	SR = −0.432***	SR = 0.481***	MW = − 0.515***
**Deprivation measures (Economic and social capacity)**						
**DipCare-Q index**	X	X	X	X	SR = −0.442***	MW = 0.389***
**MatDCQ (material)**	SR = − 0.322***	SR = 0.236**	SR = − 0.376***	X	SR = − 0.323***	MW = 0.422***
**SocDCQ (social)**	n.s. *p* = 0.718	SR = 0.221**	SR = − 0.204**	X	SR = − 0.306***	MW = 0.156*
**Q1 DiPCare**	X	MW = 0.287***	MW = −0.361***	X	MW = − 0.317***	Phi = 0.325***
**Q3 DiPCare**	X	MW = 0.262***	MW = −0.323***	X	MW = − 0.276***	Phi = 0.389***
**Quality of life measures**						
**EQ index score**	X	X	X	X	X	MW = 0.343***
**EQ mobility**	X	X	X	X	X	n.s. *p* = 0.136
**EQ pers care**	X	X	X	X	X	MW = 0.347***
**EQ activity**	X	SR = 0.358***	SR = −0.389***	SR = 0.328***	X	MW = 0.350***
**EQ pain**	X	X	X	X	X	MW = 0.181*
**EQ mood**	X	SR = 0.419***	SR = −0.487***	SR = 0.469***	X	MW = 0.404***

*SR* Spearmans’ rank effect size, *MW* Mann-Whitney U effect size, *Phi* Chi-square effect size, *n.s.* non-significant

All results to 3 s.f. *p < 0.05 **p < 0.01 ***p < 0.001

Previously observed relationships between the DiPCare-Q (SR = − 0.229, *p* = 0.002) and MTBQ-2 (MW = − 0.362, *p* = 0.000) and younger age were confirmed, and between the DBIS and older age (SR = 0.158, *p* = 0.035). Female gender was significantly correlated to treatment burden (*p* = 0.005, Phi = 0.216). Contrary to expectation, living in a capital city (based on postcode data) was associated with higher treatment burden (*p* = 0.000), but this was not significant after controlling for age, with younger participants (who reported higher treatment burden) overrepresented in the urban setting.

We explored the influence of condition type on treatment burden. The presence of diabetes [20, 47] or a mental health condition [29] have been previously associated with treatment burden and although we noted significant correlations with the GMTBQ (diabetes *p* = 0.005; mental health *p* = 0.001), only mental health conditions remained significant when treatment burden was dichotomised (mental health *p* = 0.000, diabetes *p* = 0.057). We were unable to analyse musculoskeletal conditions because almost all participants reported this, but neither cardiovascular (*p* = 0.557) nor respiratory (*p* = 0.737) conditions were significantly correlated to treatment burden.

Low to moderate correlations (MW = 0.299 to − 0.515, *p* = 0.000) were observed between MTBQ-2 and the four self-report scales (DiPCare-Q, DBIS, PMCSMS-4 and EQ-5D5L). Since one aim was to reduce the number of questions asked, we also conducted bivariate analysis on individual EQ-5D5L questions, selecting the two questions with the greatest effect size (Q3: Activity and 5: Mood) to use in regression (Q2; Personal care was excluded because of its high floor effect). We also analysed the material and social components of the DiPCare-Q separately, which had moderate (MatDCQ: *p* = 0.000, MW = 0.422) and weak (SocDCQ: *p* = 0.033, MW = 0.156) correlations with treatment burden, as well as analysing Q1 (difficulty paying bills) and Q3 (forgoing healthcare due to cost) of the DipCare-Q in isolation. These two questions were selected because they were the most frequently endorsed, and question one alone has previously been found to predict the risk of forgoing healthcare due to cost [49].

### Multivariate analysis

#### Missing data

We undertook imputation using median score or commonest category for eleven surveys that were missing data from a single independent variable. Since the MTBQ-2 variable had almost equal numbers in each category, imputation was not undertaken for the fourteen surveys (8%) with greater than 50% of their MTBQ responses missing. These surveys were excluded from the regression analysis.

#### Multivariate modelling

We trialled several multivariate models, using the MTBQ-2 as the dependent variable, aiming to find the most parsimonious model with the best fit. All models included sex, age and one or more variables from each capacity category and the EQ questions. All variables selected were those which correlated significantly to MTBQ-2 in bivariate analysis, with the exception of the presence of diabetes, which was included because of its known association with treatment burden in other studies. First, we entered the following variables using the Forward Stepwise Wald method: sex; mental health; age; DBIS; PMCSMS-4; EQ activity; EQ mood; MatDCQ; SocDCQ. We then trialled a series of models entering the variables manually, starting with sex, age, DBIS and PMCSMS-4 and sequentially adding in different deprivation and EQ-5D5L variables to identify suitable models. Age, self-efficacy and material deprivation remained significant in every model. Hosmer-Lemeshow testing was non-significant for all models. Nagelkerke r^2^, % correct classification, sensitivity and specificity varied 2% or less between models. Models were compared using the Akaike information criterion (AIC) and Bayesian information criterion (BIC), with the final model selected having the lowest AIC and BIC scores, indicating the best fit of all models trialled. This model consisted of the following covariates: age, sex, PMCSMS-4, EQ activity and MatDCQ. Logistic regression results for this model are displayed in Table 3.

**Table 3:** Logistic regression

Variable	S.E.	2-tailed sig.	Odds ratio	95% CI lower	95% CI upper
Age	.014	0.042	0.973	0.947	0.999
Sex	.578	0.053	0.326	0.105	1.013
PMCSMS	.078	0.000	0.720	0.618	0.839
Mat DCQ	.233	0.005	1.920	1.215	3.032
EQ activity	.217	0.032	1.591	1.040	2.433

Nagelkerke *r*^2^ = 0.507

AIC = 164.079

BIC = 182.858

In the final model, age (*p* = 0.042), PMCSMS-4 (*p* = 0.000), EQ activity (*p* = 0.032) and MatDCQ (*p* = 0.005) remained significant. The model correctly classified 80.5% of cases, with sensitivity of 79.1% and specificity of 81.9%. It explained 50.7% of the variance in treatment burden (Nagelkerke *r*^2^ = 0.507). The factors having the greatest impact on treatment burden were material deprivation, EQ activity score, and self-efficacy. Odds ratios indicated that each unit increase in the 4-level MatDCQ doubled the risk of high treatment burden (92% increase) and each unit increase in the 5-level EQ activity led to a 59% increase in the risk of high treatment burden. Conversely, each point increase in PMCSMS (scored in 16 increments) was associated with a 28% reduction in the risk of high treatment burden. Examination of residuals identified only 4 outliers, most of whom had borderline GMTBQ scores just above or below the dichotomous cut-off between high and ‘other’ treatment burden.

## Discussion

This study aimed to explore the correlations between established self-report tools that measure aspects of capacity, and perceived treatment burden. We found that material deprivation, self-efficacy, usual activity level and younger age remained significant in multivariate analysis and accounted for more than half the variation in the risk of having high treatment burden.

### Relationship to other research

In our survey, both deprivation and treatment burden scores differed from previous population studies. Our DiPCare-Q index score mean was higher than previously reported (1.96 compared to 1.2) [38]. We questioned whether the impact of Covid-19 on social isolation might be contributing to this difference. However, after comparing our participant responses to previous studies, we found triple the number of positive responses to material deprivation questions in our population, but little difference in social deprivation responses. This may relate to the younger mean age of the population (with material deprivation known to be higher in younger age groups) [38], differing social welfare systems between countries, and/or sampling bias.

High treatment burden scores were also reported by 51.5% of our sample, compared to 27% in a previous study [29]. Again, the younger mean age may partially explain this, given the consistent association between younger age and higher burden [29, 39, 47]. To reflect the Australian healthcare environment, we included the financial burden question (excluded by Duncan et al), which was endorsed as at least ‘somewhat difficult’ by 54% of our population and may have resulted in a higher overall score. The MTBQ section of the survey was also not completed by 8% of participants, and these participants reported fewer mental health conditions, lower scores on the DipCare-Q and DBIS and higher EQ-5D5L scores than the rest of the population. These participants may have considered the MTBQ to be irrelevant, potentially increasing the representation of people experiencing high treatment burden.

Consistent with other literature [29, 38, 43], we also found that the MTBQ, DBIS, DiPCare-Q were all correlated with the EQ-5D5L with moderate effect sizes. Previous relationships between deprivation, treatment burden and younger age, and with the DBIS and older age, were also confirmed.

### Key findings in this study

Low self-efficacy and material deprivation were strongly associated with high perceived treatment burden, regardless of the model trialled. Both these factors have been previously correlated with high treatment burden [20, 23, 47, 50, 51] although the relationship with deprivation appears to depend on whether subjective or objective (e.g. income, area data) measures are used [29]. Since the MTBQ measures patient perceptions of treatment burden, a subjective report of deprivation (such as the DiPCare-Q) may be more sensitive than traditional measures of socioeconomic status [31]. The significance of self-efficacy and material deprivation is unsurprising since both factors are known to be strong predictors of self-management ability, treatment engagement and adherence [1, 10, 52]. Importantly, they are also closely related to each other, with financial strain and low socioeconomic status consistently associated with low self-efficacy across a range of health behaviours [53, 54]. This relationship may make it more difficult to reduce treatment burden if both low self-efficacy and material deprivation are present.

Even though disease count or severity are often used by clinicians to estimate treatment burden, we found that neither disease burden (as measured by DBIS) nor specific conditions (presence of mental health diagnosis or diabetes) remained significant after multivariate modelling. In other studies, disease burden has been associated with treatment burden [29], but relationships between treatment burden and specific conditions have been much less consistent [29, 39, 47, 55]. This again highlights how patient perception of non-medical factors (e.g., confidence in one’s abilities, available resources) may be more important than a specific diagnosis in assessing treatment burden.

In our study, age and/or EQ activity score may have moderated the influence of the DBIS in multivariate analysis. Despite older people reporting a higher DBIS score and higher disease count, treatment burden declined with age. The inverse relationship between age and treatment burden is consistent across several studies [20, 29, 47]. Younger people are likely to have greater demands on their time (work, caring responsibilities), different expectations regarding health, fewer governmental social/health provisions, and greater financial insecurity [20, 29, 56], all of which may contribute to increased treatment burden.

EQ activity (which rates perceived ability to undertake ‘work, study, housework, family or leisure activities’) was the only physical capacity measure that remained significant, suggesting that the impact of CHCs on function may be more important in terms of perceived treatment burden than the conditions themselves. This makes sense since loss of function is likely to make many treatment tasks (e.g., attending appointments, lifestyle changes, relying on family) more difficult, and may particularly relate to non-life threatening conditions that impair function such as musculoskeletal disorders, reported by 91% of our sample.

### Identifying risk of high treatment burden

The secondary aim of our study was to make progress toward developing a tool to identify those at risk of high treatment burden. Our results showed that a small number of variables, taken from three established and validated self-report scales, can explain a considerable proportion of perceived treatment burden. The results also suggest that (perceived) material deprivation, self-efficacy and usual activity levels may be more important than diagnosis or condition count. The self-report measures we used are simple and quick to use and could be easily incorporated into a clinic environment. We expect that additional capacity measures, not explored in this study, will provide further explanation for perceived treatment burden. For example, we did not assess life workload since we were unable to identify any validated self-report measures; nor did we assess resilience due to debate over whether the available measures adequately capture the concept [57]. Since our self-complete survey assumed reading skills, we were unable to include a literacy or health literacy measure despite health literacy being a known contributor to treatment burden [55, 58].

Our current results have provided us with an initial foundation. The intent is to further develop the screening tool in a larger population using additional capacity measures, including a format (e.g. phone or face-face) that allows individual literacy/health literacy to be explored.

### Strengths and limitations

Since this was a cross-sectional study, we were unable to infer causal relationships. The study did suffer from sampling bias, and the multiple modes of data collection may have resulted in two different populations. Using the internet across patient self-help groups provided a convenience sample that overrepresented women, people with musculoskeletal conditions, and possibly those who had greater health concerns. The survey was undertaken during the height of the COVID pandemic which compromised our recruitment strategy and may have impacted the low response rate (20%) for our postal survey, since we were unable to follow up non-respondents. It is also possible that the unique pressures associated with the COVID pandemic influenced participant survey responses, especially in relation to perceived treatment burden, deprivation, and quality of life. However, although the population may have been non-representative, it did report high levels of deprivation. The strong association of deprivation with multimorbidity, poorer condition trajectory and lower quality of life [59–61] means that this is an important group to study.

The use of the DiPCare-Q may be a limitation since it has not been validated in an Australian population and has previously been conducted as a phone questionnaire, although the low level of non-completion suggests that it was acceptable to participants who may be more comfortable answering questions about deprivation anonymously.

The key strengths of this study were in using already validated scales and running several models to explore how they could be combined to create a capacity measure.

## Conclusions

The ability to identify those at risk of high treatment burden may help to target support where it is most needed. Our study suggests that having a specific health condition is less important than younger age, material deprivation, low self-efficacy, and functional limitations. Recognising those who are struggling most with treatment burden is important because effective management may reduce future condition escalation and overall burden of disease.

## Supplementary Information

**Additional file 1.**

**Additional file 2.**

## Data Availability

The datasets generated and analysed during the current study are available via figshare at: 10.26181/615cdea12551c

## Abbreviations

CHC: Chronic health condition
MTBQ: Multimorbidity Treatment Burden Questionnaire
DiPCare-Q: Deprivation in Primary Care Questionnaire
MatDCQ: Material deprivation index
SocDCQ: Social deprivation index
PMCSMS-4: Short-form Perceived Medical Condition Self-Management Scale
DBIS: Disease Burden Impact Scale
SR: Spearmans’ rank effect size
MW: Mann-Whitney U effect size
AIC: Akaike Information Criterion
BIC: Bayesian Information Criterion
