# Supplementary material for: Exploring the ability of self-report measures to identify risk of high treatment burden in chronic disease patients: a cross-sectional study
Source: BMC Public Health. 2022 Jan 24;22:163. doi: 10.1186/s12889-022-12579-1 (PMC8785389; doi:10.1186/s12889-022-12579-1)
Supplement: Supplementary file 1 — Additional file 1. [file 12889_2022_12579_MOESM1_ESM.docx]

**Survey Participants Needed:**

**Measuring capacity in chronic health conditions**

Researchers at La Trobe University and Sunraysia Community Health Services are seeking volunteers to complete a short survey about how people cope with their chronic health conditions. This research will be used to help healthcare professionals to better support their clients.

**Who is the survey for?**

The survey is for anyone who:

- Is over 18 years
- Has a chronic health condition (such as diabetes, arthritis, back pain, COPD, cardiovascular disease).

**How do I take part in the study?**

If you are interested in participating, please:

- Read the attached participant information and consent form
- Complete the survey (approx. 15 minutes)
- Return it to us in the supplied reply-paid envelope.

The survey is anonymous – no personal details are recorded.

If you would like more information, please contact:

| **Name:** | Dr Evelien Spelten |
| --- | --- |
| **School/Department** | La Trobe Rural Health School |
| **Email:** | E.Spelten@latrobe.edu.au |
| **Phone:** | 03 5051 4071 |
| **Ethics Approval Number** | HEC19517 |

| **Participation Information and consent form**  The research is being carried out in partial fulfilment of a PhD under the supervision of Dr Evelien Spelten and Dr Steve Begg. The following researchers will be conducting the study: | | |
| --- | --- | --- |
| **Role** | **Name** | **Organisation** |
| Chief Investigator | Dr Evelien Spelten | LaTrobe University |
| Postgraduate Student | Ms Ruth Hardman | LaTrobe University/SCHS |
| Co-investigator | Dr Steve Begg | LaTrobe University |
| Co-investigator | Dr Kelly Naess | SCHS |
| **Research funder** | This research is supported by in-kind support by La Trobe University and SCHS | |

**What is the study about?**

You are invited to participate in a study that looks at how peoples’ daily life demands, including work, family, health and finances, affects their ability to manage chronic health conditions. We hope to find out whether people who need extra support in managing their health can be more easily identified.

**Do I have to participate?**

Being part of this study is voluntary. If you want to be part of the study we ask that you read the information below carefully and ask us any questions.

You can read the information below and decide at the end if you do not want to participate. If you decide not to participate this won’t affect your relationship with La Trobe University or any other listed organisation.

**Who is being asked to participate?**

Anyone who has one or more chronic health conditions (e.g. arthritis, diabetes, chronic pain, heart or lung disease) that impact on their day-to-day life.

**What will I be asked to do?**

If you want to take part in this study, we will ask you to complete a brief survey. It will take approximately 10-15 minutes of your time.

**What are the benefits?**

There are no direct benefits to you in taking part in this study. The expected benefits to society in general is that by identifying people who might have difficulties managing their health, we can provide support earlier and prevent health decline.

**What are the risks?**

With any study there are (1) risks we know about, (2) risks we don’t know about and (3) risks we don’t expect. If you experience something that you aren’t sure about, please contact us immediately so we can discuss the best way to manage your concerns.

| **Name/Organisation** | **Position** | **Telephone** | **Email** |
| --- | --- | --- | --- |
| Dr Evelien Spelten | Chief Investigator | 03 5051 4071 | E.Spelten@latrobe.edu.au |

We have listed the risks we know about below. This will help you decide if you want to be part of the study.

- Some people experience distress when thinking about the difficulties in managing their health.
- We don’t expect any other risks associated with this study.

**What will happen to information about me?**

We will **collect** information about you in ways that will not reveal who you are.

We will **store** information about you in ways that will not reveal who you are.

We will **publish** information about you in ways that will not be identified in any type of publication from this study.

We will **keep** your information for 7 years after the project is completed. After this time we will destroy all of your data.

The storage, transfer and destruction of your data will be undertaken in accordance with the [Research Data Management Policy](https://policies.latrobe.edu.au/document/view.php?id=106/) <https://policies.latrobe.edu.au/document/view.php?id=106/>.

The personal information you provide will be handled in accordance with applicable privacy laws, any health information collected will be handled in accordance with the Health Records Act 2001 (Vic). Subject to any exceptions in relevant laws, you have the right to access and correct your personal information by contacting the research team.

**Will I hear about the results of the study?**

The study results may be published in professional journals. We cannot let you know individually about the results of the study since we are not recording your contact details.

**What if I change my mind?**

If you no longer want to complete the questionnaire, don’t return it to us. If you change your mind after sending in the survey, we cannot withdraw your responses because we cannot link who you are with your questionnaire responses.

Your decision to withdraw at any point will **not** affect your relationship with La Trobe University or any other organisation listed.

**Who can I contact for questions or want more information?**

If you would like to speak to us, please use the contact details below:

| **Name/Organisation** | **Position** | **Telephone** | **Email** |
| --- | --- | --- | --- |
| Dr Evelien Spelten | Chief Investigator | 03 5051 4071 | E.Spelten@latrobe.edu.au |

**What if I have a complaint?**

If you have a complaint about any part of this study, please contact:

| **Ethics Reference Number** | **Position** | **Telephone** | **Email** |
| --- | --- | --- | --- |
| HEC 19517 | Senior Research Ethics Officer | +61 3 9479 1443 | [humanethics@latrobe.edu.au](mailto:humanethics@latrobe.edu.au) |

**Consent Form – Declaration by Participant**

I (the participant) have read and understood the Participant Information Statement, and any questions have been answered to my satisfaction. I agree to participate in the study. I agree information provided by me or with my permission during the project may be included in a thesis, presentation and published in journals on the condition that I cannot be identified.

I would like my information collected for this research study to be:

Only used for this specific study.

**I agree, start questionnaire**

**This survey is designed to help us understand the challenges people face when living with chronic health conditions. First, we would like some general information about you and the health conditions you have.**

**Please check the box or fill in the blank as indicated.**

**Your age in years:__________________**

**Your sex:**

- **Male**
- **Female**
- **Prefer not to say**

**Your employment status (tick all relevant boxes):**

- **Employed full-time**
- **Employed part-time**
- **Looking for work**
- **Not working due to health**
- **Home duties or full-time carer**
- **Retired**
- **Student**
- **Other:________________________**

**Do you have any of the following conditions?**[1]

If yes, indicate how much each condition interferes with your daily activities by circling the number.

1 = no impact on daily activities; 5 = severe impact on daily activities

**Only tick the box if you have the condition.**

| **Do you have..** | **Amount of interference** | | | | |
| --- | --- | --- | --- | --- | --- |
| - Angina/heart disease | 1 | 2 | 3 | 4 | 5 |
| - High blood pressure | 1 | 2 | 3 | 4 | 5 |
| - High cholesterol | 1 | 2 | 3 | 4 | 5 |
| - Heart failure | 1 | 2 | 3 | 4 | 5 |
| - Poor circulation | 1 | 2 | 3 | 4 | 5 |
| - Back pain/sciatica | 1 | 2 | 3 | 4 | 5 |
| - Osteoarthritis | 1 | 2 | 3 | 4 | 5 |
| - Osteoporosis | 1 | 2 | 3 | 4 | 5 |
| - Rheumatoid arthritis | 1 | 2 | 3 | 4 | 5 |
| - Other pain problem | 1 | 2 | 3 | 4 | 5 |
| - Overweight | 1 | 2 | 3 | 4 | 5 |
| - Vision problem (apart from glasses) | 1 | 2 | 3 | 4 | 5 |
| - Hearing problem/deafness | 1 | 2 | 3 | 4 | 5 |
| - Diabetes | 1 | 2 | 3 | 4 | 5 |
| - Cancer (in last 5 years) | 1 | 2 | 3 | 4 | 5 |
| - Stroke | 1 | 2 | 3 | 4 | 5 |
| - Neurological condition (e.g. MS, Parkinsons, epilepsy) | 1 | 2 | 3 | 4 | 5 |
| - Gut problems (e.g. gastritis, reflux) | 1 | 2 | 3 | 4 | 5 |
| - Bowel problems (e.g. IBS, diverticulitis) | 1 | 2 | 3 | 4 | 5 |
| - Asthma | 1 | 2 | 3 | 4 | 5 |
| - Bronchitis /COPD | 1 | 2 | 3 | 4 | 5 |
| - Depression/anxiety | 1 | 2 | 3 | 4 | 5 |
| - Other mental health (eg Bipolar,PTSD) | 1 | 2 | 3 | 4 | 5 |
| - Thyroid problems | 1 | 2 | 3 | 4 | 5 |
| - Kidney disease | 1 | 2 | 3 | 4 | 5 |
| - Liver disease | 1 | 2 | 3 | 4 | 5 |
| - Other: | 1 | 2 | 3 | 4 | 5 |
| - Other: | 1 | 2 | 3 | 4 | 5 |
| - Other: | 1 | 2 | 3 | 4 | 5 |

These questions are about how much your illness and/or its treatment interferes with different aspects of your life. The scale is numbered from 0 to 7. 0 means there is **no** interference. 7 means there is **severe** interference. Circle the box to best describe your current life situation.[2]

**How much does your illness and/or its treatment interfere with…**

|  | **Not at all Severely** | | | | | | | | **Not applicable** |
| --- | --- | --- | --- | --- | --- | --- | --- | --- | --- |
| Your feeling of being healthy? | 0 | 1 | 2 | 3 | 4 | 5 | 6 | 7 |  |
| The things you eat and drink? | 0 | 1 | 2 | 3 | 4 | 5 | 6 | 7 |  |
| Your work, including jobs, chores or errands? | 0 | 1 | 2 | 3 | 4 | 5 | 6 | 7 |  |
| Playing sports, gardening, other physical recreation or hobbies? | 0 | 1 | 2 | 3 | 4 | 5 | 6 | 7 |  |
| Quiet recreation or hobbies such as reading, TV, music, knitting etc.? | 0 | 1 | 2 | 3 | 4 | 5 | 6 | 7 |  |
| Your financial situation? | 0 | 1 | 2 | 3 | 4 | 5 | 6 | 7 |  |
| Your relationship with your spouse or domestic partner? | 0 | 1 | 2 | 3 | 4 | 5 | 6 | 7 |  |
| Your sex life? | 0 | 1 | 2 | 3 | 4 | 5 | 6 | 7 |  |
| Your relationship and social activities with your family? | 0 | 1 | 2 | 3 | 4 | 5 | 6 | 7 |  |
| Social activities with your friends, neighbours or groups? | 0 | 1 | 2 | 3 | 4 | 5 | 6 | 7 |  |
| Your religious or spiritual activities? | 0 | 1 | 2 | 3 | 4 | 5 | 6 | 7 |  |
| Your involvement in civic or community activities? | 0 | 1 | 2 | 3 | 4 | 5 | 6 | 7 |  |
| Your self-improvement or self-expression activities? | 0 | 1 | 2 | 3 | 4 | 5 | 6 | 7 |  |

**The next four statements relate to the confidence you have in your ability to manage your health conditions. How strongly do you agree or disagree with the following? Tick the box that applies to you.** [3]

I succeed in the projects I undertake to manage my health conditions.

- Strongly agree
- Somewhat agree
- Neither agree or disagree
- Somewhat disagree
- Strongly disagree

Typically, my plans for managing my conditions don’t work out well.

- Strongly agree
- Somewhat agree
- Neither agree or disagree
- Somewhat disagree
- Strongly disagree

No matter how hard I try, managing my health conditions doesn’t turn out the way I would like.

- Strongly agree
- Somewhat agree
- Neither agree or disagree
- Somewhat disagree
- Strongly disagree

I’m generally able to accomplish my goals with respect to managing my health conditions.

- Strongly agree
- Somewhat agree
- Neither agree or disagree
- Somewhat disagree
- Strongly disagree

**The following questions ask about the treatments you undertake and the things that health providers ask you to do.**[4]

Please tell us about **the things that you do to look after your health** and how this affects your daily life. **How much difficulty** do you have with the following? (tick the box that most applies to you)

|  | Extremely difficult | Very difficult | Quite difficult | A little difficult | Not difficult | **Not applicable** |
| --- | --- | --- | --- | --- | --- | --- |
| Taking lots of medications |  |  |  |  |  |  |
| Remembering how and when to take medication |  |  |  |  |  |  |
| Paying for prescriptions, over-the-counter medication or equipment |  |  |  |  |  |  |
| Collecting prescription medication |  |  |  |  |  |  |
| Monitoring your medical conditions (e.g. checking blood pressure or blood sugar, monitoring symptoms). |  |  |  |  |  |  |
| Arranging appointments with health professionals |  |  |  |  |  |  |
| Seeing lots of health professionals |  |  |  |  |  |  |
| Attending appointments with health professionals (e.g. time off work, arranging transport etc.) |  |  |  |  |  |  |
| Getting healthcare in the evenings and weekends. |  |  |  |  |  |  |
| Getting help from community services (e.g. physiotherapy, district nursing etc.) |  |  |  |  |  |  |
| Obtaining clear and up-to-date information about your condition |  |  |  |  |  |  |
| Making recommended lifestyle changes (e.g. diet, exercise etc.) |  |  |  |  |  |  |
| Having to rely on help from family and friends |  |  |  |  |  |  |

Many people report difficulty in following all the recommendations given by their healthcare providers. How often was each of the following statements true for you **over the past 4 weeks?**[5]. Tick the box that applies to you.

1. **I had a hard time doing what my health providers suggested I do.**

- None of the time
- A little of the time
- Some of the time
- A good bit of the time
- Most of the time
- All of the time.

1. **I followed my health providers instructions exactly.**

- None of the time
- A little of the time
- Some of the time
- A good bit of the time
- Most of the time
- All of the time.

1. **I was unable to do what was necessary to follow my health providers’ treatment plans.**

- None of the time
- A little of the time
- Some of the time
- A good bit of the time
- Most of the time
- All of the time.

1. **I found it easy to do the things my health providers suggested I do**.

- None of the time
- A little of the time
- Some of the time
- A good bit of the time
- Most of the time
- All of the time.

1. **Overall, how often were you able to do what your health providers told you to do?**

- None of the time
- A little of the time
- Some of the time
- A good bit of the time
- Most of the time
- All of the time.

Living with chronic conditions often leads to high levels of financial strain and social isolation. To better understand this, we would appreciate your answers to the following questions about your personal finances, social environment and general health. Please tick the box that applies to your situation.[6]

| **During the last 12 months,** have you had trouble paying your household bills? (tax, insurance, phone, electricity, credit cards etc?) | **Yes** | **No** |
| --- | --- | --- |
| **During the last 12 months,** have you had to ask your immediate family for money to cover your basic day-to-day needs? | **Yes** | **No** |
| **During the last 12 months,** has a member of your household not sought treatment (dentist, doctor, buying medication) because you didn’t have enough money? | **Yes** | **No** |
| **During the last 12 months,** have you feared being evicted from or losing your home? | **Yes** | **No** |
| **During the last 12 months,** have you not bought clothes even though you or a member of your household needed them? | **Yes** | **No** |
| **During the last 12 months,** have you not bought furniture or household goods even though you or a member of your household needed them? | **Yes** | **No** |
| **During the last 12 months,** have you gone on holiday? | **Yes** | **No** |
| **During the last 3 months,** have you spent an evening in the company of close family members or friends? | **Yes** | **No** |
| **During the last 3 months,** have you been to the cinema, theatre, a concert or a sports event? | **Yes** | **No** |
| **During the last month,** has there been an occasion when your household did not have enough to eat? | **Yes** | **No** |
| **During the last month,** have you been able to access the internet (at home, work, at a library, internet café etc)? | **Yes** | **No** |
| If you’re in difficulty, is there someone **outside your household** to whom you can turn for material help (money, food accommodation)? | **Yes** | **No** |
| **Are you currently** finding it very difficult to pay back money (to the bank, family etc)? | **Yes** | **No** |
| **Do you currently** suffer from a physical disability that has a major impact on your day-to-day life? | **Yes** | **No** |
| **Do you currently** suffer from mental health issues or problems that have a major impact on your day-to-day life? | **Yes** | **No** |
| **Do you currently** have problems linked to alcohol consumption, drug-taking, gambling etc.? | **Yes** | **No** |
| Deprivation in Primary Care Questionnaire: Vaucher P, Bischoff T, Diserens EA, Herzig L, Meystre-Agustoni G, Panese F, Favrat B, Sass C, Bodenmann P. Detecting and measuring deprivation in primary care: development, reliability and validity of a self-reported questionnaire: the DiPCare-Q. BMJ Open. 2012 Feb 3;2(1):e000692. | | |

| **EQ-5D quality of life questions**[7]  Under each heading, please tick the ONE box that best describes your health TODAY. | |
| --- | --- |
| MOBILITY |  |
| I have no problems with walking around | ❑ |
| I have slight problems with walking around | ❑ |
| I have moderate problems with walking around | ❑ |
| I have severe problems with walking around | ❑ |
| I am unable to walk around | ❑ |
| PERSONAL CARE |  |
| I have no problems with washing or dressing myself | ❑ |
| I have slight problems with washing or dressing myself | ❑ |
| I have moderate problems with washing or dressing myself | ❑ |
| I have severe problems with washing or dressing myself | ❑ |
| I am unable to wash or dress myself | ❑ |
| USUAL ACTIVITIES *(e.g. work, study, housework, family or leisure activities)* |  |
| I have no problems doing my usual activities | ❑ |
| I have slight problems doing my usual activities | ❑ |
| I have moderate problems doing my usual activities | ❑ |
| I have severe problems doing my usual activities | ❑ |
| I am unable to do my usual activities | ❑ |
| PAIN / DISCOMFORT |  |
| I have no pain or discomfort | ❑ |
| I have slight pain or discomfort | ❑ |
| I have moderate pain or discomfort | ❑ |
| I have severe pain or discomfort | ❑ |
| I have extreme pain or discomfort | ❑ |
| ANXIETY / DEPRESSION |  |
| I am not anxious or depressed | ❑ |
| I am slightly anxious or depressed | ❑ |
| I am moderately anxious or depressed | ❑ |
| I am severely anxious or depressed | ❑ |
| I am extremely anxious or depressed | ❑ |

The best health you can imagine

| We would like to know how good or bad your health is TODAY. |
| --- |
| This scale is numbered from 0 to 100. |
| 100 means the best health you can imagine. 0 means the worst health you can imagine. |
| Mark an X on the scale to indicate how your health is TODAY. |
| Now, please write the number you marked on the scale in the box below. |

YOUR HEALTH TODAY =

10

0

20

30

40

50

60

80

70

90

100

5

15

25

35

45

55

75

65

85

95

The worst health you can imagine

**Thank you for completing the survey.**

Please return it to SCHS in the envelope provided.

Information about support services

Some people experience distress after thinking about their health and associated difficulties in managing it. If this is the case for you, please seek support from family members, trusted friends, your GP or other mental health support services you have used in the past.

The following 24-hour phone services can also provide support:

Lifeline: 13 11 14

Nurse-On-Call: 1300 60 60 24

References

1. Bayliss, E.A., J.L. Ellis, and J.F. Steiner, *Subjective assessments of comorbidity correlate with quality of life health outcomes: Initial validation of a comorbidity assessment instrument.* Health and Quality of Life Outcomes, 2005. **3**(1): p. 51.

2. Devins, G.M., *Using the illness intrusiveness ratings scale to understand health-related quality of life in chronic disease.* J Psychosom Res, 2010. **68**(6): p. 591-602.

3. Wild, M.G., et al., *Validation of the shortened Perceived Medical Condition Self-Management Scale in patients with chronic disease.* Psychol Assess, 2018. **30**(10): p. 1300-1307.

4. Duncan, P., et al., *Development and validation of the Multimorbidity Treatment Burden Questionnaire (MTBQ).* BMJ Open, 2018. **8**(4): p. e019413.

5. Kravitz, R.L., et al., *Recall of Recommendations and Adherence to Advice Among Patients With Chronic Medical Conditions.* Arch Intern Med, 1993. **153**: p. 1869-1878

6. Vaucher, P., et al., *Detecting and measuring deprivation in primary care: development, reliability and validity of a self-reported questionnaire: the DiPCare-Q.* BMJ Open, 2012. **2**(1): p. e000692.

7. Herdman, M., et al., *Development and preliminary testing of the new five-level version of EQ-5D (EQ-5D-5L).* An International Journal of Quality of Life Aspects of Treatment, Care and Rehabilitation - Official Journal of the International Society of Quality of Life Research, 2011. **20**(10): p. 1727-1736.
